# Supplementary material for: Chargaff’s second parity rule lies at the origin of additive genetic interactions in quantitative traits to make omnigenic selection possible
Source: PeerJ. 2023 Dec 14;11:e16671. doi: 10.7717/peerj.16671 (PMC10725672; doi:10.7717/peerj.16671)
Supplement: Supplemental Information 1 [file peerj-11-16671-s001.pdf]

Supporting Information

Tables S1-2 and S4-5

Chargaff's second parity rule lies at the origin of additive genetic interactions in quantitative traits to make omnigenetic selection possible

Bakhyt T. Matkarimov[1,2,]* and Murat K. Saparbaev[3,4,]*.

[1] *National Laboratory Astana, Nazarbayev University, Astana 010000, Kazakhstan.*

[2] *L.N.Gumilev Eurasian National University, Astana 010000, Kazakhstan.*

[3] *Groupe «Mechanisms of DNA Repair and Carcinogenesis», CNRS UMR9019, Université Paris-Saclay, Gustave Roussy Cancer Campus, F-94805 Villejuif Cedex, France.*

[4] *Al-Farabi Kazakh National University, Almaty, Kazakhstan.*

*To whom correspondence should be addressed: M.K. Saparbaev. **E-mail**: murat.saparbaev@gustaveroussy.fr

Correspondence may also be addressed to B.T. Matkarimov. **E-mail**: bmatkarimov@nu.edu.kz

Supplementary Table S1. Mutation spectra inferred from single nucleotide polymorphisms in human and mice populations.

N°	N X N	N Y N	X	Y	Total number of SNPs	Fraction	N’X’N’	N’Y’N’	X’	Y’	Total number of SNPs	Fraction	Difference (%)
	**Human SNPs**												
1	AGC	ATC	G	T	312720	0.37	GAT	GCT	A	C	310244	0.36	0.79
2	CAA	CTA	A	T	227826	0.27	TAG	TTG	A	T	229630	0.27	0.79
3	TCT	TGT	C	G	902323	1.06	ACA	AGA	C	G	895602	1.05	0.74
4	CGT	CTT	G	T	296420	0.35	AAG	ACG	A	C	294421	0.34	0.67
5	TAT	TGT	A	G	3028959	3.54	ACA	ATA	C	T	3010412	3.52	0.61
6	AGT	ATT	G	T	472582	0.55	AAT	ACT	A	C	469735	0.55	0.60
7	CCT	CGT	C	G	379974	0.44	ACG	AGG	C	G	377686	0.44	0.60
8	CCC	CGC	C	G	306967	0.36	GCG	GGG	C	G	305208	0.36	0.57
9	GAT	GTT	A	T	427135	0.50	AAC	ATC	A	T	424703	0.50	0.57
10	AAA	ACA	A	C	887037	1.04	TGT	TTT	G	T	891542	1.04	0.51
11	GAC	GCC	A	C	377851	0.44	GGC	GTC	G	T	379592	0.44	0.46
12	TGA	TTA	G	T	494282	0.58	TAA	TCA	A	C	496509	0.58	0.45
13	AAG	ATG	A	T	441016	0.52	CAT	CTT	A	T	442585	0.52	0.35
14	ACT	ATT	C	T	2367625	2.77	AAT	AGT	A	G	2375298	2.78	0.32
15	GAC	GGC	A	G	1039990	1.22	GCC	GTC	C	T	1036758	1.21	0.31
16	ACG	ATG	C	T	3502135	4.10	CAT	CGT	A	G	3511739	4.11	0.27
17	TCC	TTC	C	T	1292114	1.51	GAA	GGA	A	G	1288797	1.51	0.26
18	AAA	AGA	A	G	1729945	2.02	TCT	TTT	C	T	1734336	2.03	0.25
19	TAT	TTT	A	T	544308	0.64	AAA	ATA	A	T	543030	0.64	0.23
20	GAG	GTG	A	T	287742	0.34	CAC	CTC	A	T	288408	0.34	0.23
21	GAA	GCA	A	C	512618	0.60	TGC	TTC	G	T	513797	0.60	0.23
22	ACC	ATC	C	T	1331428	1.56	GAT	GGT	A	G	1334457	1.56	0.23
23	GCA	GTA	C	T	1234387	1.44	TAC	TGC	A	G	1237171	1.45	0.23
24	GAG	GCG	A	C	240273	0.28	CGC	CTC	G	T	240790	0.28	0.21
25	TCC	TGC	C	G	444969	0.52	GCA	GGA	C	G	444029	0.52	0.21
26	GGG	GTG	G	T	467854	0.55	CAC	CCC	A	C	468761	0.55	0.19
27	GGA	GTA	G	T	367737	0.43	TAC	TCC	A	C	368387	0.43	0.18
28	TGG	TTG	G	T	543832	0.64	CAA	CCA	A	C	542895	0.64	0.17
29	CAA	CGA	A	G	1798724	2.10	TCG	TTG	C	T	1801266	2.11	0.14
30	CGA	CTA	G	T	160741	0.19	TAG	TCG	A	C	160957	0.19	0.13
31	TCG	TGG	C	G	280491	0.33	CCA	CGA	C	G	280147	0.33	0.12
32	GCT	GTT	C	T	1378880	1.61	AAC	AGC	A	G	1380364	1.62	0.11
33	CAG	CCG	A	C	292541	0.34	CGG	CTG	G	T	292773	0.34	0.08
34	TAC	TTC	A	T	230424	0.27	GAA	GTA	A	T	230244	0.27	0.08

35	AGA	ATA	G	T	604514	0.71	TAT	TCT	A	C	604874	0.71	0.06
36	CCG	CTG	C	T	2588225	3.03	CAG	CGG	A	G	2589637	3.03	0.05
37	GGT	GTT	G	T	611030	0.71	AAC	ACC	A	C	611362	0.72	0.05
38	AAG	AGG	A	G	1664646	1.95	CCT	CTT	C	T	1663799	1.95	0.05
39	CAT	CCT	A	C	481839	0.56	AGG	ATG	G	T	482018	0.56	0.04
40	CCA	CTA	C	T	1383378	1.62	TAG	TGG	A	G	1383081	1.62	0.02
41	GCT	GGT	C	G	418689	0.49	ACC	AGC	C	G	418602	0.49	0.02
42	GAG	GGG	A	G	1195336	1.40	CCC	CTC	C	T	1195523	1.40	0.02
43	GCG	GTG	C	T	2008008	2.35	CAC	CGC	A	G	2008195	2.35	0.01
44	TAA	TGA	A	G	1448254	1.69	TCA	TTA	C	T	1448196	1.69	0.00
45	TAA	TTA	A	T	532422	0.62							
46	CAG	CTG	A	T	343352	0.40							
47	GCC	GGC	C	G	364797	0.43							
48	ACT	AGT	C	G	690362	0.81							
49	CCG	CGG	C	G	135675	0.16							
50	AAT	ATT	A	T	533699	0.62							
51	TCA	TGA	C	G	596062	0.70							
52	GAC	GTC	A	T	256117	0.30							
N°	N X N	N Y N	X	Y	Total number of SNPs	Fraction	N’X’N’	N’Y’N’	X’	Y’	Total number of SNPs	Fraction	Difference (%)
	**Mouse SNPs**												
1	CGA	CTA	G	T	38698	0.26	TAG	TCG	A	C	38235	0.25	1.20
2	AAG	ATG	A	T	88927	0.59	CAT	CTT	A	T	89884	0.59	1.06
3	CCC	CGC	C	G	29680	0.20	GCG	GGG	C	G	29385	0.19	0.99
4	CCT	CGT	C	G	48777	0.32	ACG	AGG	C	G	49266	0.32	0.99
5	AGA	ATA	G	T	107141	0.71	TAT	TCT	A	C	108135	0.71	0.92
6	GAC	GCC	A	C	57560	0.38	GGC	GTC	G	T	57041	0.38	0.90
7	TGA	TTA	G	T	86691	0.57	TAA	TCA	A	C	87432	0.58	0.85
8	GCT	GTT	C	T	323720	2.14	AAC	AGC	A	G	326448	2.15	0.84
9	AGC	ATC	G	T	85493	0.56	GAT	GCT	A	C	84799	0.56	0.81
10	CAA	CTA	A	T	54492	0.36	TAG	TTG	A	T	54163	0.36	0.60
11	TCG	TGG	C	G	38824	0.26	CCA	CGA	C	G	39042	0.26	0.56
12	GCT	GGT	C	G	65256	0.43	ACC	AGC	C	G	64903	0.43	0.54
13	TGG	TTG	G	T	88219	0.58	CAA	CCA	A	C	88685	0.58	0.53
14	GGA	GTA	G	T	66022	0.44	TAC	TCC	A	C	66361	0.44	0.51
15	TCT	TGT	C	G	120710	0.80	ACA	AGA	C	G	121326	0.80	0.51
16	TAC	TTC	A	T	60192	0.40	GAA	GTA	A	T	60497	0.40	0.50
17	AAA	ACA	A	C	151052	1.00	TGT	TTT	G	T	150333	0.99	0.48
18	CGT	CTT	G	T	75731	0.50	AAG	ACG	A	C	75412	0.50	0.42

19	CCG	CTG	C	T	348208	2.30	CAG	CGG	A	G	349621	2.31	0.40
20	GAG	GCG	A	C	50929	0.34	CGC	CTC	G	T	50724	0.33	0.40
21	CAG	CCG	A	C	56636	0.37	CGG	CTG	G	T	56410	0.37	0.40
22	GAT	GTT	A	T	63675	0.42	AAC	ATC	A	T	63432	0.42	0.38
23	ACG	ATG	C	T	480989	3.17	CAT	CGT	A	G	482775	3.18	0.37
24	GGG	GTG	G	T	74901	0.49	CAC	CCC	A	C	74624	0.49	0.37
25	GCG	GTG	C	T	276960	1.83	CAC	CGC	A	G	277835	1.83	0.31
26	GAG	GGG	A	G	225182	1.49	CCC	CTC	C	T	224605	1.48	0.26
27	GAA	GCA	A	C	95996	0.63	TGC	TTC	G	T	95759	0.63	0.25
28	TCC	TGC	C	G	56075	0.37	GCA	GGA	C	G	55960	0.37	0.21
29	TCC	TTC	C	T	351509	2.32	GAA	GGA	A	G	352103	2.32	0.17
30	AAA	AGA	A	G	362257	2.39	TCT	TTT	C	T	361808	2.39	0.12
31	GAG	GTG	A	T	73085	0.48	CAC	CTC	A	T	72996	0.48	0.12
32	CCA	CTA	C	T	283060	1.87	TAG	TGG	A	G	282755	1.87	0.11
33	ACC	ATC	C	T	262283	1.73	GAT	GGT	A	G	262546	1.73	0.10
34	CAT	CCT	A	C	83574	0.55	AGG	ATG	G	T	83647	0.55	0.09
35	AGT	ATT	G	T	100114	0.66	AAT	ACT	A	C	100033	0.66	0.08
36	GGT	GTT	G	T	95675	0.63	AAC	ACC	A	C	95744	0.63	0.07
37	ACT	ATT	C	T	384248	2.53	AAT	AGT	A	G	384516	2.54	0.07
38	TAA	TGA	A	G	285259	1.88	TCA	TTA	C	T	285425	1.88	0.06
39	GAC	GGC	A	G	253253	1.67	GCC	GTC	C	T	253112	1.67	0.06
40	TAT	TGT	A	G	477219	3.15	ACA	ATA	C	T	477431	3.15	0.04
41	GCA	GTA	C	T	255518	1.69	TAC	TGC	A	G	255481	1.69	0.01
42	TAT	TTT	A	T	103335	0.68	AAA	ATA	A	T	103323	0.68	0.01
43	AAG	AGG	A	G	299963	1.98	CCT	CTT	C	T	299979	1.98	0.01
44	CAA	CGA	A	G	264331	1.74	TCG	TTG	C	T	264323	1.74	0.00
45	TAA	TTA	A	T	133002	0.88							
46	CAG	CTG	A	T	84745	0.56							
47	GCC	GGC	C	G	49385	0.33							
48	ACT	AGT	C	G	107564	0.71							
49	CCG	CGG	C	G	34929	0.23							
50	AAT	ATT	A	T	122244	0.81							
51	TCA	TGA	C	G	65934	0.43							
52	GAC	GTC	A	T	52841	0.35							

Supplementary Table S2. Mutation spectra inferred from single nucleotide insertion or deletion (indels) in the genomes in human populations.

N°	N Δ N	N X N	Δ	X	Total number of indels	Fraction	N' Δ N'	N'X'N'	Δ	X'	Total number of indels	Fraction	Difference (%)
	Triplets with sequence context in which a base corresponding to indel is different from the neighboring base.												
1	C-G	CTG	-	T	31093	1.74	C-G	CAG	-	A	31132	1.75	0.13
2	G-C	GTC	-	T	6049	0.34	G-C	GAC	-	A	6092	0.34	0.71
3	T-C	TGC	-	G	11496	0.64	G-A	GCA	-	C	11346	0.64	1.30
4	T-T	TAT	-	A	33846	1.90	A-A	ATA	-	T	33349	1.87	1.47
5	T-T	TGT	-	G	44135	2.48	A-A	ACA	-	C	43138	2.42	2.26
6	T-C	TAC	-	A	7640	0.43	G-A	GTA	-	T	7449	0.42	2.50
7	C-A	CTA	-	T	16203	0.91	T-G	TAG	-	A	15787	0.89	2.57
8	A-C	ATC	-	T	11841	0.66	G-T	GAT	-	A	11511	0.65	2.79
9	A-T	AGT	-	G	22001	1.23	A-T	ACT	-	C	22719	1.27	3.16
10	A-G	ATG	-	T	17041	0.96	C-T	CAT	-	A	16374	0.92	3.91
11	G-T	GCT	-	C	15314	0.86	A-C	AGC	-	G	14649	0.82	4.34
12	A-A	AGA	-	G	36802	2.06	T-T	TCT	-	C	38617	2.17	4.70
13	G-G	GAG	-	A	16611	0.93	C-C	CTC	-	T	17504	0.98	5.10
14	C-C	CGC	-	G	2900	0.16	G-G	GCG	-	C	3063	0.17	5.32
15	C-C	CAC	-	A	13485	0.76	G-G	GTG	-	T	12680	0.71	5.97
16	A-G	ACG	-	C	4610	0.26	C-T	CGT	-	G	4249	0.24	7.83
17	T-G	TCG	-	C	2880	0.16	C-A	CGA	-	G	2603	0.15	9.62
18	T-A	TGA	-	G	16897	0.95	T-A	TCA	-	C	19855	1.11	14.90
	Triplets with sequence context in which a base corresponding to indel is the same as neighboring base.												
19	A-A	AAA	-	A	19326	1.08	T-T	TTT	-	T	19540	1.10	1.10
20	G-G	GGG	-	G	1144	0.06	C-C	CCC	-	C	1124	0.06	1.75
21	T-G	TTG	-	T	11150	0.63	C-A	CAA	-	A	123998	6.95	91.01
22	C-T	CTT	-	T	138611	7.77	A-G	AAG	-	A	11140	0.62	91.96
23	A-C	AAC	-	A	4327	0.24	G-T	GTT	-	T	79624	4.47	94.57
24	T-A	TTA	-	T	6518	0.37	T-A	TAA	-	A	133014	7.46	95.10
25	A-T	ATT	-	T	155454	8.72	A-T	AAT	-	A	7175	0.40	95.38
26	T-C	TTC	-	T	5072	0.28	G-A	GAA	-	A	112956	6.34	95.51
27	C-G	CCG	-	C	337	0.02	C-G	CGG	-	G	14700	0.82	97.71
28	G-T	GGT	-	G	1290	0.07	A-C	ACC	-	C	58855	3.30	97.81
29	T-C	TCC	-	C	62091	3.48	G-A	GGA	-	G	1287	0.07	97.93
30	T-G	TGG	-	G	73594	4.13	C-A	CCA	-	C	1510	0.08	97.95
31	C-T	CCT	-	C	1435	0.08	A-G	AGG	-	G	72608	4.07	98.02
32	G-C	GCC	-	C	41325	2.32	G-C	GGC	-	G	750	0.04	98.19

Supplementary Materials Table S4

Analysis of the nucleotide composition at different codon positions in CDSs of human and mouse by taking in account orientation of the genes relative to reference strand

We processed CDS data from the curated database, CCDS for human/mouse [33] (*Supplementary Materials*, Table S3). We computed statistics/counts for nucleotide sequences of the length 1 (single nucleotides), 2 (dinucleotides) and 3 (trinucleotides) started at the 1st, 2nd and 3rd codon positions for two cases: properly for CDS sequences and for the CDS sequences read from a single strand, e.g. reverse complementary CDS for the portion of proteins, located on different strand. For the reverse complementary CDSs we setup 1st, 2nd and 3rd codon positions relative to the 5′→3′ DNA stand orientation. In addition, we compute the total count as a sum of counts for all codon positions. To compare statistical counts for nucleotide sequences and their reverse complementary counterparts we have used percentage difference formula 200*(A-B)/(A+B). In a single row of the supplementary data files we show the name of the sequence (SEQ), its counts at the first (P1), second (P2), third (P3) codon positions and total count (TOTAL); then the same data for reverse complementary sequence; then percentage difference for 1st, 2nd and 3rd codon positions and total between sequence and it reverse complementary (for more details see *Supplementary Materials*, Table S4).

Mononucleotide statistics and comparison with reverse-complemetary counterpart at the three codon positions for CDS data of the Consensus coding sequence (CCDS) database

Homo sapiens, CCDS v.24, CDS counts, strand orientation not taken in account (strand-independent case)

SEQ	P1	P2	P3	TOTAL	RC SE	P1	P2	P3	TOTAL	diff P1	diff P2	diff P3	diff
A	5606547	6574757	4173307	16354611	T	3510236	5374049	4715804	13600089	45.98795	20.09754	-12.20588	18.39125
C	5093123	4885610	5983004	15961737	G	6521808	3897298	5859599	16278705	-24.60083	22.50535	2.084086	-1.966276
G	6521808	3897298	5859599	16278705	C	5093123	4885610	5983004	15961737	24.60083	-22.50535	-2.084086	1.966276
T	3510236	5374049	4715804	13600089	A	5606547	6574757	4173307	16354611	-45.98795	-20.09754	12.20588	-18.39125

Homo sapiens, CCDS v.24, Watson (+) strand counts, CDS on Crick (-) strand reverse complemented (strand-dependent case)

SEQ	P1	P2	P3	TOTAL	RC SE	P1	P2	P3	TOTAL	diff P1	diff P2	diff P3	diff
A	4569047	5977430	4436912	14983389	T	4547736	5971376	4452199	14971311	0.467511	0.101332	-0.343949	0.080642
C	5802969	4403887	5932901	16139757	G	5811962	4379021	5909702	16100685	-0.154852	0.566236	0.391789	0.242379
G	5811962	4379021	5909702	16100685	C	5802969	4403887	5932901	16139757	0.154852	-0.566236	-0.391789	-0.242379
T	4547736	5971376	4452199	14971311	A	4569047	5977430	4436912	14983389	-0.467511	-0.101332	0.343949	-0.080642

Mus musculus, CCDS v.23, CDS counts, strand orientation not taken in account (strand-independent case)

SEQ	P1	P2	P3	TOTAL	RC SE	P1	P2	P3	TOTAL	diff P1	diff P2	diff P3	diff
A	4067939	4694120	2919241	11681300	T	2583958	3967677	3392355	9943990	44.61828	16.77349	-14.9919	16.06739
C	3690518	3528000	4425972	11644490	G	4670524	2823142	4275371	11769037	-23.4422	22.19626	3.461558	-1.063889
G	4670524	2823142	4275371	11769037	C	3690518	3528000	4425972	11644490	23.4422	-22.19626	-3.461558	1.063889
T	2583958	3967677	3392355	9943990	A	4067939	4694120	2919241	11681300	-44.61828	-16.77349	14.9919	-16.06739

Mus musculus, CCDS v.23, Watson (+) strand counts, CDS on Crick (-) strand reverse complemented (strand-dependent case)

SEQ	P1	P2	P3	TOTAL	RC SE	P1	P2	P3	TOTAL	diff P1	diff P2	diff P3	diff
A	3329925	4342707	3158599	10831231	T	3321972	4319090	3152997	10794059	0.23912	0.545314	0.177515	0.343783
C	4179281	3176278	4341371	11696930	G	4181761	3174864	4359972	11716597	-0.059323	0.044527	-0.427543	-0.167997
G	4181761	3174864	4359972	11716597	C	4179281	3176278	4341371	11696930	0.059323	-0.044527	0.427543	0.167997
T	3321972	4319090	3152997	10794059	A	3329925	4342707	3158599	10831231	-0.23912	-0.545314	-0.177515	-0.343783

Dinucleotide statistics and comparison with reverse-complemetary counterpart at the three codon positions for CDS data of the Consensus coding sequence (CCDS) database

Homo sapiens, CCDS v.24, CDS counts, strand orientation not taken in account (strand-independent case)

SEQ	P1	P2	P3	TOTAL	RC SE(	P1	P2	P3	TOTAL	diff P1	diff P2	diff P3	diff
AA	1964658	1482979	1109002	4556639	TT	1180438	1207693	785147	3173278	49.86938	20.46225	34.1953	35.79239
AC	1107286	1514092	819751	3441129	GT	1238739	799200	775597	2813536	-11.20644	61.80733	5.535344	20.068
AG	1179410	2244374	1626701	5050485	CT	1599679	1375599	1359377	4334655	-30.24509	47.99898	17.90469	15.25454
AT	1355193	1333312	590110	3278615	AT	1355193	1333312	590110	3278615	0	0	0	0
CA	1532987	1305504	2123618	4962109	TG	714891	2067071	2079443	4861405	72.78829	-45.16235	2.102039	2.050264
CC	1295246	1705689	1717871	4718806	GG	1337786	1049336	2033520	4420642	-3.231256	47.6477	-16.82837	6.52477
CG	665211	498818	782138	1946167	CG	665211	498818	782138	1946167	0	0	0	0
CT	1599679	1375599	1359377	4334655	AG	1179410	2244374	1626701	5050485	30.24509	-47.99898	-17.90469	-15.25454
GA	2509894	755708	1528223	4793825	TC	1047689	1470169	1041029	3558887	82.20216	-64.19591	37.92497	29.56975
GC	1435389	1293054	1514428	4242871	GC	1435389	1293054	1514428	4242871	0	0	0	0
GG	1337786	1049336	2033520	4420642	CC	1295246	1705689	1717871	4718806	3.231256	-47.6477	16.82837	-6.52477
GT	1238739	799200	775597	2813536	AC	1107286	1514092	819751	3441129	11.20644	-61.80733	-5.535344	-20.068
TA	567218	629116	810185	2006519	TA	567218	629116	810185	2006519	0	0	0	0
TC	1047689	1470169	1041029	3558887	GA	2509894	755708	1528223	4793825	-82.20216	64.19591	-37.92497	-29.56975
TG	714891	2067071	2079443	4861405	CA	1532987	1305504	2123618	4962109	-72.78829	45.16235	-2.102039	-2.050264
TT	1180438	1207693	785147	3173278	AA	1964658	1482979	1109002	4556639	-49.86938	-20.46225	-34.1953	-35.79239

Homo sapiens, CCDS v.24, Watson (+) strand counts, CDS on Crick (-) strand reverse complemented (strand-dependent case)

SEQ	P1	P2	P3	TOTAL	RC SE(	P1	P2	P3	TOTAL	diff P1	diff P2	diff P3	diff
AA	1380912	1324077	1158535	3863524	TT	1151052	1577804	1137537	3866393	18.15666	-17.48707	1.829037	-0.074231
AC	942887	1384431	807614	3134932	GT	1032978	949229	1137526	3119733	-9.119145	37.29781	-33.92167	0.486005
AG	1266551	1928242	1497372	4692165	CT	1618696	1275102	1799177	4692975	-24.41004	40.77864	-18.31036	-0.017261
AT	978697	1340680	959238	3278615	AT	978697	1340680	959238	3278615	0	0	0	0
CA	1807413	1011526	2097829	4916768	TG	1405892	1807770	1693084	4906746	24.99115	-56.48531	21.35343	0.204041
CC	1657525	1533834	1392483	4583842	GG	1517335	1170227	1868044	4555606	8.831256	26.8934	-29.17081	0.617893
CG	719330	583425	643412	1946167	CG	719330	583425	643412	1946167	0	0	0	0
CT	1618696	1275102	1799177	4692975	AG	1266551	1928242	1497372	4692165	24.41004	-40.77864	18.31036	0.017261
GA	1785675	893113	1499668	4178456	TC	1285396	1988095	900765	4174256	32.5801	-76.00854	49.89958	0.100566

GC	1475962	1366452	1400457	4242871	GC	1475962	1366452	1400457	4242871	0	0	0	0
GG	1517335	1170227	1868044	4555606	CC	1657525	1533834	1392483	4583842	-8.831256	-26.8934	29.17081	-0.617893
GT	1032978	949229	1137526	3119733	AC	942887	1384431	807614	3134932	9.119145	-37.29781	33.92167	-0.486005
TA	687999	597707	720813	2006519	TA	687999	597707	720813	2006519	0	0	0	0
TC	1285396	1988095	900765	4174256	GA	1785675	893113	1499668	4178456	-32.5801	76.00854	-49.89958	-0.100566
TG	1405892	1807770	1693084	4906746	CA	1807413	1011526	2097829	4916768	-24.99115	56.48531	-21.35343	-0.204041
TT	1151052	1577804	1137537	3866393	AA	1380912	1324077	1158535	3863524	-18.15666	17.48707	-1.829037	0.074231

Mus musculus, CCDS v.23, CDS counts, strand orientation not taken in account (strand-independent case)

SEQ	P1	P2	P3	TOTAL	RC SE(	P1	P2	P3	TOTAL	diff P1	diff P2	diff P3	diff
AA	1392020	973972	717358	3083350	TT	865840	843913	536901	2246654	46.60874	14.30883	28.77508	31.3957
AC	808600	1154774	604702	2568076	GT	913367	601226	607149	2121742	-12.16829	63.04647	-0.403845	19.03417
AG	872989	1658365	1188585	3719939	CT	1194140	1040207	1051976	3286323	-31.07218	45.81371	12.19418	12.37796
AT	994330	907009	387932	2289271	AT	994330	907009	387932	2289271	0	0	0	0
CA	1108503	943112	1605070	3656685	TG	530029	1530568	1521392	3581989	70.60881	-47.49652	5.352888	2.063803
CC	921507	1202238	1239904	3363649	GG	953756	743995	1431523	3129274	-3.439411	47.09025	-14.34582	7.219399
CG	466368	342443	529022	1337833	CG	466368	342443	529022	1337833	0	0	0	0
CT	1194140	1040207	1051976	3286323	AG	872989	1658365	1188585	3719939	31.07218	-45.81371	-12.19418	-12.37796
GA	1773789	553688	1163332	3490809	TC	768281	1144727	778696	2691704	79.10939	-69.59889	39.61179	25.85049
GC	1029612	924233	1067193	3021038	GC	1029612	924233	1067193	3021038	0	0	0	0
GG	953756	743995	1431523	3129274	CC	921507	1202238	1239904	3363649	3.439411	-47.09025	14.34582	-7.219399
GT	913367	601226	607149	2121742	AC	808600	1154774	604702	2568076	12.16829	-63.04647	0.403845	-19.03417
TA	419808	448469	555366	1423643	TA	419808	448469	555366	1423643	0	0	0	0
TC	768281	1144727	778696	2691704	GA	1773789	553688	1163332	3490809	-79.10939	69.59889	-39.61179	-25.85049
TG	530029	1530568	1521392	3581989	CA	1108503	943112	1605070	3656685	-70.60881	47.49652	-5.352888	-2.063803
TT	865840	843913	536901	2246654	AA	1392020	973972	717358	3083350	-46.60874	-14.30883	-28.77508	-31.3957

Mus musculus, CCDS v.23, Watson (+) strand counts, CDS on Crick (-) strand reverse complemented (strand-dependent case)

SEQ	P1	P2	P3	TOTAL	RC SE(	P1	P2	P3	TOTAL	diff P1	diff P2	diff P3	diff
AA	966856	924427	783025	2674308	TT	789479	1114676	751541	2655696	20.19854	-18.66007	4.10331	0.698386
AC	705613	1036156	603986	2345755	GT	757576	707548	878939	2344063	-7.102705	37.6908	-37.08252	0.072156
AG	968180	1426573	1116741	3511494	CT	1185373	956867	1352528	3494768	-20.17067	39.41412	-19.09772	0.477459

AT	689276	955551	644444	2289271	AT	689276	955551	644444	2289271	0	0	0	0
CA	1316431	739942	1564123	3620496	TG	1068856	1317931	1231391	3618178	20.75851	-56.17344	23.80471	0.064045
CC	1179109	1074995	989727	3243831	GG	1097641	831353	1320098	3249092	7.156517	25.56113	-28.60572	-0.162053
CG	498366	404474	434993	1337833	CG	498366	404474	434993	1337833	0	0	0	0
CT	1185373	956867	1352528	3494768	AG	968180	1426573	1116741	3511494	20.17067	-39.41412	19.09772	-0.477459
GA	1278296	661668	1159316	3099280	TC	965558	1449648	668027	3083233	27.87508	-74.64349	53.77086	0.519109
GC	1048235	974295	998508	3021038	GC	1048235	974295	998508	3021038	0	0	0	0
GG	1097641	831353	1320098	3249092	CC	1179109	1074995	989727	3243831	-7.156517	-25.56113	28.60572	0.162053
GT	757576	707548	878939	2344063	AC	705613	1036156	603986	2345755	7.102705	-37.6908	37.08252	-0.072156
TA	484770	436835	502038	1423643	TA	484770	436835	502038	1423643	0	0	0	0
TC	965558	1449648	668027	3083233	GA	1278296	661668	1159316	3099280	-27.87508	74.64349	-53.77086	-0.519109
TG	1068856	1317931	1231391	3618178	CA	1316431	739942	1564123	3620496	-20.75851	56.17344	-23.80471	-0.064045
TT	789479	1114676	751541	2655696	AA	966856	924427	783025	2674308	-20.19854	18.66007	-4.10331	-0.698386

Trinucleotide statistics and comparison with reverse-complemetary counterpart at the three codon positions for CDS data of the Consensus coding sequence (CCDS) database

Homo sapiens, CCDS v.24, CDS counts, strand orientation not taken in account (strand-independent case)

SEQ	P1	P2	P3	TOTAL	RC SEQ	P1	P2	P3	TOTAL	diff P1	diff P2	diff P3	diff
AAA	539355	424075	423518	1386948	TTT	354871	214282	292020	861173	41.26116	65.72905	36.755	46.77462
AAC	389409	275755	205418	870582	GTT	235841	127023	245053	607917	49.12211	73.85309	-17.59714	35.53131
AAG	669186	565470	232326	1466982	CTT	279736	232274	438024	950034	82.08262	83.53457	-61.37033	42.77572
AAT	366708	207656	247740	822104	ATT	337245	211568	205336	754149	8.370729	-1.866305	18.71827	8.622347
ACA	324102	539981	274131	1138214	TGT	212724	282328	425323	920375	41.49501	62.66574	-43.23143	21.16391
ACC	376567	436288	227855	1040710	GGT	222142	122070	355702	699914	51.586	112.5507	-43.81646	39.15791
ACG	122179	213224	85177	420580	CGT	94504	66589	181612	342705	25.54423	104.8093	-72.29309	20.40522
ACT	284438	324599	232588	841625	AGT	269830	304610	276096	850536	5.271096	6.353692	-17.1061	-1.053209
AGA	257732	653669	681463	1592864	TCT	326602	348994	299354	974950	-23.57214	60.77316	77.91647	48.12763
AGC	409311	575263	350189	1334763	GCT	385770	299491	505292	1190553	5.921661	63.05133	-36.261	11.42114
AGG	242537	703001	318953	1264491	CCT	378789	386293	562401	1327483	-43.85846	58.14922	-55.24409	-4.860543
AGT	269830	304610	276096	850536	ACT	284438	324599	232588	841625	-5.271096	-6.353692	17.1061	1.053209
ATA	159174	259298	96842	515314	TAT	251787	102144	212522	566453	-45.07143	86.95946	-74.78569	-9.454716
ATC	412049	247212	178772	838033	GAT	482989	94326	321045	898360	-15.85184	89.52796	-56.93004	-6.948542
ATG	446725	615234	109160	1171119	CAT	231828	185984	538370	956182	63.33978	107.1494	-132.5684	20.20748
ATT	337245	211568	205336	754149	AAT	366708	207656	247740	822104	-8.370729	1.866305	-18.71827	-8.622347
CAA	270722	278127	646578	1195427	TTG	269581	468404	108247	846232	0.422356	-50.97632	142.6373	34.20699
CAC	305454	267263	444513	1017230	GTG	561286	377222	175000	1113508	-59.03316	-34.12306	87.00802	-9.037057
CAG	724983	574130	494157	1793270	CTG	789479	618583	322484	1730546	-8.517348	-7.454098	42.04369	3.560004
CAT	231828	185984	538370	956182	ATG	446725	615234	109160	1171119	-63.33978	-107.1494	132.5684	-20.20748
CCA	370730	626530	499193	1496453	TGG	245487	782801	481928	1510216	40.64899	-22.17662	3.519444	-0.915498
CCC	404738	462705	398091	1265534	GGG	325944	336742	356085	1018771	21.56725	31.51253	11.13957	21.60508
CCG	140989	230161	258186	629336	CGG	235368	210976	180820	627164	-50.15398	8.697978	35.24599	0.345722
CCT	378789	386293	562401	1327483	AGG	242537	703001	318953	1264491	43.85846	-58.14922	55.24409	4.860543
CGA	131932	68587	249598	450117	TCG	90722	155439	121527	367688	37.01708	-77.53743	69.01772	20.1586
CGC	203407	152666	170108	526181	GCG	144928	183314	200321	528563	33.5763	-18.24394	-16.31244	-0.451674
CGG	235368	210976	180820	627164	CCG	140989	230161	258186	629336	50.15398	-8.697978	-35.24599	-0.345722
CGT	94504	66589	181612	342705	ACG	122179	213224	85177	420580	-25.54423	-104.8093	72.29309	-20.40522
CTA	149040	194638	222740	566418	TAG	7832	205140	135143	348115	180.0296	-5.253916	48.95287	47.74087

CTC	381424	330104	376129	1087657	GAG	842373	281961	317784	1442118	-75.33096	15.73134	16.81623	-28.02312
CTG	789479	618583	322484	1730546	CAG	724983	574130	494157	1793270	8.517348	7.454098	-42.04369	-3.560004
CTT	279736	232274	438024	950034	AAG	669186	565470	232326	1466982	-82.08262	-83.53457	61.37033	-42.77572
GAA	662879	222350	605650	1490879	TTC	388601	302634	264828	956063	52.16989	-30.58531	78.30686	43.71301
GAC	521653	139351	283744	944748	GTC	288095	161079	227960	677134	57.68659	-14.4646	21.80323	33.00043
GAG	842373	281961	317784	1442118	CTC	381424	330104	376129	1087657	75.33096	-15.73134	-16.81623	28.02312
GAT	482989	94326	321045	898360	ATC	412049	247212	178772	838033	15.85184	-89.52796	56.93004	6.948542
GCA	341475	421122	438577	1201174	TGC	238899	508370	427708	1174977	35.34824	-18.77327	2.509336	2.204995
GCC	563216	389127	370238	1322581	GGC	441437	278129	487384	1206950	24.243	33.26999	-27.3188	9.142485
GCG	144928	183314	200321	528563	CGC	203407	152666	170108	526181	-33.5763	18.24394	16.31244	0.451674
GCT	385770	299491	505292	1190553	AGC	409311	575263	350189	1334763	-5.921661	-63.05133	36.261	-11.42114
GGA	348263	312395	834349	1495007	TCC	361168	429751	299062	1089981	-3.638127	-31.62612	94.45594	31.33678
GGC	441437	278129	487384	1206950	GCC	563216	389127	370238	1322581	-24.243	-33.26999	27.3188	-9.142485
GGG	325944	336742	356085	1018771	CCC	404738	462705	398091	1265534	-21.56725	-31.51253	-11.13957	-21.60508
GGT	222142	122070	355702	699914	ACC	376567	436288	227855	1040710	-51.586	-112.5507	43.81646	-39.15791
GTA	153517	133876	127584	414977	TAC	297576	137382	173608	608566	-63.87109	-2.584993	-30.56124	-37.82723
GTC	288095	161079	227960	677134	GAC	521653	139351	283744	944748	-57.68659	14.4646	-21.80323	-33.00043
GTG	561286	377222	175000	1113508	CAC	305454	267263	444513	1017230	59.03316	34.12306	-87.00802	9.037057
GTT	235841	127023	245053	607917	AAC	389409	275755	205418	870582	-49.12211	-73.85309	17.59714	-35.53131
TAA	10023	184450	288912	483385	TTA	167385	222373	120052	509810	-177.4012	-18.64349	82.5794	-5.321211
TAC	297576	137382	173608	608566	GTA	153517	133876	127584	414977	63.87109	2.584993	30.56124	37.82723
TAG	7832	205140	135143	348115	CTA	149040	194638	222740	566418	-180.0296	5.253916	-48.95287	-47.74087
TAT	251787	102144	212522	566453	ATA	159174	259298	96842	515314	45.07143	-86.95946	74.78569	9.454716
TCA	269197	535985	321086	1126268	TGA	17781	493572	744484	1255837	175.2162	8.239078	-79.46883	-10.87853
TCC	361168	429751	299062	1089981	GGA	348263	312395	834349	1495007	3.638127	31.62612	-94.45594	-31.33678
TCG	90722	155439	121527	367688	CGA	131932	68587	249598	450117	-37.01708	77.53743	-69.01772	-20.1586
TCT	326602	348994	299354	974950	AGA	257732	653669	681463	1592864	23.57214	-60.77316	-77.91647	-48.12763
TGA	17781	493572	744484	1255837	TCA	269197	535985	321086	1126268	-175.2162	-8.239078	79.46883	10.87853
TGC	238899	508370	427708	1174977	GCA	341475	421122	438577	1201174	-35.34824	18.77327	-2.509336	-2.204995
TGG	245487	782801	481928	1510216	CCA	370730	626530	499193	1496453	-40.64899	22.17662	-3.519444	0.915498
TGT	212724	282328	425323	920375	ACA	324102	539981	274131	1138214	-41.49501	-62.66574	43.23143	-21.16391
TTA	167385	222373	120052	509810	TAA	10023	184450	288912	483385	177.4012	18.64349	-82.5794	5.321211
TTC	388601	302634	264828	956063	GAA	662879	222350	605650	1490879	-52.16989	30.58531	-78.30686	-43.71301
TTG	269581	468404	108247	846232	CAA	270722	278127	646578	1195427	-0.422356	50.97632	-142.6373	-34.20699

TTT	354871	214282	292020	861173	AAA	539355	424075	423518	1386948	-41.26116	-65.72905	-36.755	-46.77462

Homo sapiens, CCDS v.24, Watson (+) strand counts, CDS on Crick (-) strand reverse complemented (strand-dependent case)

SEQ	P1	P2	P3	TOTAL	RC SE	P1	P2	P3	TOTAL	diff P1	diff P2	diff P3	diff
AAA	376132	357321	388676	1122129	TTT	390372	318591	417029	1125992	-3.715571	11.46007	-7.03806	-0.343665
AAC	262068	257841	221202	741111	GTT	256201	166294	314893	737388	2.264075	43.1688	-34.95313	0.503619
AAG	453541	496813	256540	1206894	CTT	423455	232758	553909	1210122	6.861149	72.38638	-73.38377	-0.267106
AAT	289171	206969	292117	788257	ATT	271530	231606	284860	787996	6.29248	-11.23502	2.515525	0.033117
ACA	302084	487839	242983	1032906	TGT	372470	280496	372717	1025683	-20.8689	53.97203	-42.14195	0.701743
ACC	252106	397206	225334	874646	GGT	325692	174890	365396	865978	-25.47119	77.71982	-47.41997	0.995965
ACG	95239	197572	90319	383130	CGT	150737	76404	153014	380155	-45.12473	88.45154	-51.53021	0.779525
ACT	293458	301809	248978	844245	AGT	297860	270314	279742	847916	-1.488877	11.00987	-11.63716	-0.433883
AGA	299262	480996	502865	1283123	TCT	487160	517089	280442	1284691	-47.78554	-7.23245	56.79076	-0.122127
AGC	357942	540336	367167	1265445	GCT	480076	325921	453874	1259871	-29.1483	49.50378	-21.12124	0.44145
AGG	311487	632577	347598	1291662	CCT	537525	357243	405544	1300312	-53.2473	55.63315	-15.3878	-0.667445
AGT	297860	270314	279742	847916	ACT	293458	301809	248978	844245	1.488877	-11.00987	11.63716	0.433883
ATA	130780	236812	174330	541922	TAT	254111	100060	185674	539845	-64.0862	81.18929	-6.302152	0.384001
ATC	257350	281320	328998	867668	GAT	367877	135331	365517	868725	-35.3558	70.07735	-10.5164	-0.121747
ATG	319037	573548	171050	1063635	CAT	422153	147750	493763	1063666	-27.82444	118.0644	-97.08384	-0.002914
ATT	271530	231606	284860	787996	AAT	289171	206969	292117	788257	-6.29248	11.23502	-2.515525	-0.033117
CAA	369266	193419	460222	1022907	TTG	275264	553984	189504	1018752	29.16916	-96.48476	83.33297	0.407022
CAC	342593	222057	502658	1067308	GTG	416581	408458	238391	1063430	-19.49171	-59.12659	71.32241	0.364005
CAG	673401	448300	641186	1762887	CTG	684955	554117	521857	1760929	-1.701174	-21.11237	20.52014	0.11113
CAT	422153	147750	493763	1063666	ATG	319037	573548	171050	1063635	27.82444	-118.0644	97.08384	0.002914
CCA	571608	560035	375702	1507345	TGG	429896	643914	425514	1499324	28.29984	-13.93398	-12.4341	0.533547
CCC	373331	411109	364208	1148648	GGG	391831	365256	378570	1135657	-4.835577	11.81223	-3.867104	1.137414
CCG	175061	205447	247029	627537	CGG	233816	233991	161156	628963	-28.73969	-12.99114	42.07553	-0.22698
CCT	537525	357243	405544	1300312	AGG	311487	632577	347598	1291662	53.2473	-55.63315	15.3878	0.667445
CGA	142293	95544	171458	409295	TCG	79756	202399	126355	408510	56.32721	-71.72848	30.28948	0.191977
CGC	192484	177486	157784	527754	GCG	148762	176720	201508	526990	25.62492	0.432517	-24.33898	0.144869
CGG	233816	233991	161156	628963	CCG	175061	205447	247029	627537	28.73969	12.99114	-42.07553	0.22698
CGT	150737	76404	153014	380155	ACG	95239	197572	90319	383130	45.12473	-88.45154	51.53021	-0.779525
CTA	177436	164953	116595	458984	TAG	100501	212939	142109	455549	55.36147	-25.39667	-19.72447	0.751203

CTC	332850	323274	606816	1262940	GAG	590487	325964	350384	1266835	-55.80563	-0.828664	53.57961	-0.307933
CTG	684955	554117	521857	1760929	CAG	673401	448300	641186	1762887	1.701174	21.11237	-20.52014	-0.11113
CTT	423455	232758	553909	1210122	AAG	453541	496813	256540	1206894	-6.861149	-72.38638	73.38377	0.267106
GAA	482198	242331	497256	1221785	TTC	309480	450038	465639	1225157	43.6334	-59.99893	6.567071	-0.275609
GAC	345113	180467	286511	812091	GTC	215232	221245	373314	809791	46.35751	-20.30211	-26.31092	0.283621
GAG	590487	325964	350384	1266835	CTC	332850	323274	606816	1262940	55.80563	0.828664	-53.57961	0.307933
GAT	367877	135331	365517	868725	ATC	257350	281320	328998	867668	35.3558	-70.07735	10.5164	0.121747
GCA	422782	425897	338504	1187183	TGC	328528	474566	385874	1188968	25.09058	-10.80977	-13.07881	-0.150243
GCC	424342	437914	406571	1268827	GGC	414100	322040	524564	1260704	2.443103	30.495	-25.34391	0.642253
GCG	148762	176720	201508	526990	CGC	192484	177486	157784	527754	-25.62492	-0.432517	24.33898	-0.144869
GCT	480076	325921	453874	1259871	AGC	357942	540336	367167	1265445	29.1483	-49.50378	21.12124	-0.44145
GGA	385712	308041	599514	1293267	TCC	338216	629424	324081	1291721	13.12175	-68.56427	59.64367	0.119614
GGC	414100	322040	524564	1260704	GCC	424342	437914	406571	1268827	-2.443103	-30.495	25.34391	-0.642253
GGG	391831	365256	378570	1135657	CCC	373331	411109	364208	1148648	4.835577	-11.81223	3.867104	-1.137414
GGT	325692	174890	365396	865978	ACC	252106	397206	225334	874646	25.47119	-77.71982	47.41997	-0.995965
GTA	144964	153229	210928	509121	TAC	216759	132879	164784	514422	-39.69612	14.2254	24.5635	-1.035814
GTC	215232	221245	373314	809791	GAC	345113	180467	286511	812091	-46.35751	20.30211	26.31092	-0.283621
GTG	416581	408458	238391	1063430	CAC	342593	222057	502658	1067308	19.49171	59.12659	-71.32241	-0.364005
GTT	256201	166294	314893	737388	AAC	262068	257841	221202	741111	-2.264075	-43.1688	34.95313	-0.503619
TAA	116628	151829	228246	496703	TTA	175936	255191	65365	496492	-40.54361	-50.78964	110.9502	0.042489
TAC	216759	132879	164784	514422	GTA	144964	153229	210928	509121	39.69612	-14.2254	-24.5635	1.035814
TAG	100501	212939	142109	455549	CTA	177436	164953	116595	458984	-55.36147	25.39667	19.72447	-0.751203
TAT	254111	100060	185674	539845	ATA	130780	236812	174330	541922	64.0862	-81.18929	6.302152	-0.384001
TCA	380264	639183	169887	1189334	TGA	274998	408794	508979	1192771	32.12944	43.96833	-99.89954	-0.288568
TCC	338216	629424	324081	1291721	GGA	385712	308041	599514	1293267	-13.12175	68.56427	-59.64367	-0.119614
TCG	79756	202399	126355	408510	CGA	142293	95544	171458	409295	-56.32721	71.72848	-30.28948	-0.191977
TCT	487160	517089	280442	1284691	AGA	299262	480996	502865	1283123	47.78554	7.23245	-56.79076	0.122127
TGA	274998	408794	508979	1192771	TCA	380264	639183	169887	1189334	-32.12944	-43.96833	99.89954	0.288568
TGC	328528	474566	385874	1188968	GCA	422782	425897	338504	1187183	-25.09058	10.80977	13.07881	0.150243
TGG	429896	643914	425514	1499324	CCA	571608	560035	375702	1507345	-28.29984	13.93398	12.4341	-0.533547
TGT	372470	280496	372717	1025683	ACA	302084	487839	242983	1032906	20.8689	-53.97203	42.14195	-0.701743
TTA	175936	255191	65365	496492	TAA	116628	151829	228246	496703	40.54361	50.78964	-110.9502	-0.042489
TTC	309480	450038	465639	1225157	GAA	482198	242331	497256	1221785	-43.6334	59.99893	-6.567071	0.275609
TTG	275264	553984	189504	1018752	CAA	369266	193419	460222	1022907	-29.16916	96.48476	-83.33297	-0.407022

TTT	390372	318591	417029	1125992	AAA	376132	357321	388676	1122129	3.715571	-11.46007	7.03806	0.343665

Mus musculus, CCDS v.23, CDS counts, strand orientation not taken in account (strand-independent case)

SEQ	P1	P2	P3	TOTAL	RC SE	P1	P2	P3	TOTAL	diff P1	diff P2	diff P3	diff
AAA	344524	259057	264355	867936	TTT	251749	144997	194324	591070	31.1183	56.4578	30.53595	37.95269
AAC	300835	187146	136101	624082	GTT	161998	90122	193590	445710	59.99443	69.98572	-34.87447	33.34704
AAG	507899	397294	161003	1066196	CTT	201310	167565	349537	718412	86.45942	81.3403	-73.8567	38.97595
AAT	238762	123133	155899	517794	ATT	228856	134217	128389	491462	4.236792	-8.61395	19.35361	5.218101
ACA	242693	417104	199596	859393	TGT	165691	226026	330440	722157	37.71059	59.42127	-49.37174	17.35462
ACC	274864	327332	165117	767313	GGT	167385	92653	261079	521117	48.60565	111.7559	-45.03186	38.21643
ACG	85561	155201	63276	304038	CGT	69528	53615	120755	243898	20.67587	97.29714	-62.46665	21.95147
ACT	205482	255137	176713	637332	AGT	198622	234855	201091	634568	3.395166	8.278503	-12.9051	0.434625
AGA	186949	496851	488735	1172535	TCT	248960	281583	239636	770179	-28.45135	55.30796	68.39893	41.42205
AGC	301964	421685	262384	986033	GCT	301236	227745	365985	894966	0.241379	59.72622	-32.97457	9.682833
AGG	185454	498800	236375	920629	CCT	284529	287511	411783	983823	-42.1611	53.74184	-54.12507	-6.63645
AGT	198622	234855	201091	634568	ACT	205482	255137	176713	637332	-3.395166	-8.278503	12.9051	-0.434625
ATA	110566	165602	63894	340062	TAT	175959	69248	146671	391878	-45.64558	82.05578	-78.6237	-14.15854
ATC	321744	170302	120153	612199	GAT	328264	65446	247504	641214	-2.006129	88.956	-69.27707	-4.629759
ATG	333164	436888	75496	845548	CAT	164024	130105	417445	711574	68.03865	108.214	-138.7383	17.2079
ATT	228856	134217	128389	491462	AAT	238762	123133	155899	517794	-4.236792	8.61395	-19.35361	-5.218101
CAA	186267	185957	484540	856764	TTG	201337	335527	77494	614358	-7.775978	-57.36322	144.8475	32.95525
CAC	227619	205586	331881	765086	GTG	414952	284707	133064	832723	-58.30733	-32.27499	85.5228	-8.466218
CAG	530593	421464	371204	1323261	CTG	581115	464270	243975	1289360	-9.089077	-9.665656	41.36325	2.595172
CAT	164024	130105	417445	711574	ATG	333164	436888	75496	845548	-68.03865	-108.214	138.7383	-17.2079
CCA	270157	460456	368717	1099330	TGG	176351	567495	353073	1096919	42.01761	-20.8257	4.334779	0.219556
CCC	272350	309867	279311	861528	GGG	224671	226640	243335	694646	19.18591	31.0255	13.76687	21.44773
CCG	94471	144404	180093	418968	CGG	157519	138588	120973	417080	-50.04008	4.110364	39.27378	0.451649
CCT	284529	287511	411783	983823	AGG	185454	498800	236375	920629	42.1611	-53.74184	54.12507	6.63645
CGA	101660	55727	174575	331962	TCG	65069	114346	91793	271208	43.89278	-68.93393	62.15611	20.1449
CGC	137661	94513	112719	344893	GCG	97342	115071	131206	343619	34.3136	-19.61791	-15.15794	0.370073
CGG	157519	138588	120973	417080	CCG	94471	144404	180093	418968	50.04008	-4.110364	-39.27378	-0.451649
CGT	69528	53615	120755	243898	ACG	85561	155201	63276	304038	-20.67587	-97.29714	62.46665	-21.95147

CTA	120389	146830	173175	440394	TAG	6175	155624	93674	255473	180.4842	-5.815099	59.58501	53.14837
CTC	291326	261542	285289	838157	GAG	613698	214203	247108	1075009	-71.24054	19.901	14.34306	-24.76021
CTG	581115	464270	243975	1289360	CAG	530593	421464	371204	1323261	9.089077	9.665656	-41.36325	-2.595172
CTT	201310	167565	349537	718412	AAG	507899	397294	161003	1066196	-86.45942	-81.3403	73.8567	-38.97595
GAA	435839	152723	451355	1039917	TTC	307106	218769	182660	708535	34.65479	-35.55716	84.75982	37.90576
GAC	395988	107994	217365	721347	GTC	224551	128083	180179	532813	55.25422	-17.01902	18.70787	30.06538
GAG	613698	214203	247108	1075009	CTC	291326	261542	285289	838157	71.24054	-19.901	-14.34306	24.76021
GAT	328264	65446	247504	641214	ATC	321744	170302	120153	612199	2.006129	-88.956	69.27707	4.629759
GCA	244744	311214	313441	869399	TGC	174623	361753	318219	854595	33.44135	-15.01976	-1.512839	1.717407
GCC	386290	270203	256561	913054	GGC	309985	189242	336290	835517	21.91806	35.24296	-26.89681	8.868613
GCG	97342	115071	131206	343619	CGC	137661	94513	112719	344893	-34.3136	19.61791	15.15794	-0.370073
GCT	301236	227745	365985	894966	AGC	301964	421685	262384	986033	-0.241379	-59.72622	32.97457	-9.682833
GGA	251715	235460	590819	1077994	TCC	268734	332502	220518	821754	-6.540122	-34.172	91.28167	26.97621
GGC	309985	189242	336290	835517	GCC	386290	270203	256561	913054	-21.91806	-35.24296	26.89681	-8.868613
GGG	224671	226640	243335	694646	CCC	272350	309867	279311	861528	-19.18591	-31.0255	-13.76687	-21.44773
GGT	167385	92653	261079	521117	ACC	274864	327332	165117	767313	-48.60565	-111.7559	45.03186	-38.21643
GTA	111866	98314	100316	310496	TAC	230332	103976	123251	457559	-69.23828	-5.597904	-20.51734	-38.29491
GTC	224551	128083	180179	532813	GAC	395988	107994	217365	721347	-55.25422	17.01902	-18.70787	-30.06538
GTG	414952	284707	133064	832723	CAC	227619	205586	331881	765086	58.30733	32.27499	-85.5228	8.466218
GTT	161998	90122	193590	445710	AAC	300835	187146	136101	624082	-59.99443	-69.98572	34.87447	-33.34704
TAA	7342	119621	191770	318733	TTA	105648	144620	82423	332691	-174.0083	-18.92136	79.75915	-4.285381
TAC	230332	103976	123251	457559	GTA	111866	98314	100316	310496	69.23828	5.597904	20.51734	38.29491
TAG	6175	155624	93674	255473	CTA	120389	146830	173175	440394	-180.4842	5.815099	-59.58501	-53.14837
TAT	175959	69248	146671	391878	ATA	110566	165602	63894	340062	45.64558	-82.05578	78.6237	14.15854
TCA	185518	416296	226749	828563	TGA	13364	375294	519660	908318	173.1218	10.3594	-78.48539	-9.183703
TCC	268734	332502	220518	821754	GGA	251715	235460	590819	1077994	6.540122	34.172	-91.28167	-26.97621
TCG	65069	114346	91793	271208	CGA	101660	55727	174575	331962	-43.89278	68.93393	-62.15611	-20.1449
TCT	248960	281583	239636	770179	AGA	186949	496851	488735	1172535	28.45135	-55.30796	-68.39893	-41.42205
TGA	13364	375294	519660	908318	TCA	185518	416296	226749	828563	-173.1218	-10.3594	78.48539	9.183703
TGC	174623	361753	318219	854595	GCA	244744	311214	313441	869399	-33.44135	15.01976	1.512839	-1.717407
TGG	176351	567495	353073	1096919	CCA	270157	460456	368717	1099330	-42.01761	20.8257	-4.334779	-0.219556
TGT	165691	226026	330440	722157	ACA	242693	417104	199596	859393	-37.71059	-59.42127	49.37174	-17.35462
TTA	105648	144620	82423	332691	TAA	7342	119621	191770	318733	174.0083	18.92136	-79.75915	4.285381
TTC	307106	218769	182660	708535	GAA	435839	152723	451355	1039917	-34.65479	35.55716	-84.75982	-37.90576

TTG	201337	335527	77494	614358	CAA	186267	185957	484540	856764	7.775978	57.36322	-144.8475	-32.95525
TTT	251749	144997	194324	591070	AAA	344524	259057	264355	867936	-31.1183	-56.4578	-30.53595	-37.95269

Mus musculus, CCDS v.23, Watson (+) strand counts, CDS on Crick (-) strand reverse complemented (strand-dependent case)

SEQ	P1	P2	P3	TOTAL	RC SE	P1	P2	P3	TOTAL	diff P1	diff P2	diff P3	diff
AAA	246485	227564	259556	733605	TTT	253898	203274	268229	725401	-2.96293	11.2757	-3.286566	1.124601
AAC	195794	191136	148747	535677	GTT	173596	113511	247008	534115	12.01873	50.96062	-49.65749	0.292019
AAG	337626	376271	181886	895783	CTT	297688	163540	427597	888825	12.57268	78.81685	-80.62932	0.779779
AAT	186951	125714	192836	505501	ATT	175370	145310	183075	503755	6.392674	-14.46071	5.193251	0.345997
ACA	234206	375873	184002	794081	TGT	288882	212122	286465	787469	-20.90509	55.69809	-43.558	0.836142
ACC	182068	293952	166321	642341	GGT	247829	128831	269429	646089	-30.59384	78.11147	-47.32438	-0.581793
ACG	69411	138253	66671	274335	CGT	112266	57886	103449	273601	-47.17713	81.94903	-43.23771	0.267915
ACT	219928	228076	186992	634996	AGT	227374	205691	203839	636904	-3.329294	10.32121	-8.621118	-0.300024
AGA	236931	368535	370900	976366	TCT	373572	380441	212335	966348	-44.76342	-3.179274	54.37431	1.031341
AGC	265467	393379	282809	941655	GCT	362009	242737	334598	939344	-30.77154	47.36306	-16.77629	0.24572
AGG	238408	455844	259193	953445	CCT	394038	260715	296254	951007	-49.21527	54.46279	-13.34457	0.256032
AGT	227374	205691	203839	636904	ACT	219928	228076	186992	634996	3.329294	-10.32121	8.621118	0.300024
ATA	89947	156384	120060	366391	TAT	169754	67046	128749	365549	-61.46068	79.96957	-6.984474	0.230074
ATC	192372	209902	222402	624676	GAT	250718	92372	285647	628737	-26.33596	77.76388	-24.8972	-0.647991
ATG	231587	430648	118907	781142	CAT	299586	102800	373594	775980	-25.60333	122.9166	-103.426	0.663018
ATT	175370	145310	183075	503755	AAT	186951	125714	192836	505501	-6.392674	14.46071	-5.193251	-0.345997
CAA	262903	132055	341038	735996	TTG	194325	409881	130920	735126	29.99729	-102.5309	89.04097	0.118277
CAC	257621	170022	372391	800034	GTG	310180	308444	179151	797775	-18.51318	-57.86075	70.07263	0.282762
CAG	496321	335065	477100	1308486	CTG	499766	418075	386294	1304135	-0.691707	-22.04371	21.03466	0.333075
CAT	299586	102800	373594	775980	ATG	231587	430648	118907	781142	25.60333	-122.9166	103.426	-0.663018
CCA	419518	405231	272622	1097371	TGG	320333	468290	310255	1098878	26.81216	-14.43789	-12.91284	-0.137234
CCC	248709	276150	252201	777060	GGG	267791	252806	258517	779114	-7.388964	8.826443	-2.473381	-0.263981
CCG	116844	132899	168650	418393	CGG	150998	159800	106857	417655	-25.5031	-18.38134	44.85766	0.176545
CCT	394038	260715	296254	951007	AGG	238408	455844	259193	953445	49.21527	-54.46279	13.34457	-0.256032
CGA	108862	73202	120096	302160	TCG	60215	143861	96934	301010	57.5442	-65.1046	21.34451	0.381319
CGC	126240	113586	104591	344417	GCG	95973	114009	134113	344095	27.24143	-0.371713	-24.73524	0.093535
CGG	150998	159800	106857	417655	CCG	116844	132899	168650	418393	25.5031	18.38134	-44.85766	-0.176545
CGT	112266	57886	103449	273601	ACG	69411	138253	66671	274335	47.17713	-81.94903	43.23771	-0.267915

CTA	137579	120099	89195	346873	TAG	76060	165820	107114	348994	57.59154	-31.98179	-18.25591	-0.609599
CTC	250340	255153	449442	954935	GAG	435955	251436	270840	958231	-54.0919	1.467462	49.59224	-0.34456
CTG	499766	418075	386294	1304135	CAG	496321	335065	477100	1308486	0.691707	22.04371	-21.03466	-0.333075
CTT	297688	163540	427597	888825	AAG	337626	376271	181886	895783	-12.57268	-78.81685	80.62932	-0.779779
GAA	328491	167532	382389	878412	TTC	228489	333895	307656	870040	35.90865	-66.35582	21.66033	0.957647
GAC	263132	143667	220440	627239	GTC	165624	174334	286963	626921	45.48414	-19.28736	-26.22097	0.050711
GAG	435955	251436	270840	958231	CTC	250340	255153	449442	954935	54.0919	-1.467462	-49.59224	0.34456
GAT	250718	92372	285647	628737	ATC	192372	209902	222402	624676	26.33596	-77.76388	24.8972	0.647991
GCA	303381	313588	244644	861613	TGC	243335	338058	280988	862381	21.96607	-7.510213	-13.82869	-0.089095
GCC	286872	303961	285153	875986	GGC	289191	222170	361224	872585	-0.80512	31.0915	-23.53766	0.389003
GCG	95973	114009	134113	344095	CGC	126240	113586	104591	344417	-27.24143	0.371713	24.73524	-0.093535
GCT	362009	242737	334598	939344	AGC	265467	393379	282809	941655	30.77154	-47.36306	16.77629	-0.24572
GGA	292830	227546	430928	951304	TCC	251054	460624	236766	948444	15.3621	-67.73849	58.15898	0.301093
GGC	289191	222170	361224	872585	GCC	286872	303961	285153	875986	0.80512	-31.0915	23.53766	-0.389003
GGG	267791	252806	258517	779114	CCC	248709	276150	252201	777060	7.388964	-8.826443	2.473381	0.263981
GGT	247829	128831	269429	646089	ACC	182068	293952	166321	642341	30.59384	-78.11147	47.32438	0.581793
GTA	108176	111257	165817	385250	TAC	163102	102496	117207	382805	-40.49425	8.197312	34.35044	0.636673
GTC	165624	174334	286963	626921	GAC	263132	143667	220440	627239	-45.48414	19.28736	26.22097	-0.050711
GTG	310180	308444	179151	797775	CAC	257621	170022	372391	800034	18.51318	57.86075	-70.07263	-0.282762
GTT	173596	113511	247008	534115	AAC	195794	191136	148747	535677	-12.01873	-50.96062	49.65749	-0.292019
TAA	75854	101473	148968	326295	TTA	112767	167626	44736	325129	-39.13986	-49.16629	107.6199	0.357985
TAC	163102	102496	117207	382805	GTA	108176	111257	165817	385250	40.49425	-8.197312	-34.35044	-0.636673
TAG	76060	165820	107114	348994	CTA	137579	120099	89195	346873	-57.59154	31.98179	18.25591	0.609599
TAT	169754	67046	128749	365549	ATA	89947	156384	120060	366391	61.46068	-79.96957	6.984474	-0.230074
TCA	280717	464722	121992	867431	TGA	216306	299461	353683	869450	25.91872	43.25168	-97.41567	-0.232486
TCC	251054	460624	236766	948444	GGA	292830	227546	430928	951304	-15.3621	67.73849	-58.15898	-0.301093
TCG	60215	143861	96934	301010	CGA	108862	73202	120096	302160	-57.5442	65.1046	-21.34451	-0.381319
TCT	373572	380441	212335	966348	AGA	236931	368535	370900	976366	44.76342	3.179274	-54.37431	-1.031341
TGA	216306	299461	353683	869450	TCA	280717	464722	121992	867431	-25.91872	-43.25168	97.41567	0.232486
TGC	243335	338058	280988	862381	GCA	303381	313588	244644	861613	-21.96607	7.510213	13.82869	0.089095
TGG	320333	468290	310255	1098878	CCA	419518	405231	272622	1097371	-26.81216	14.43789	12.91284	0.137234
TGT	288882	212122	286465	787469	ACA	234206	375873	184002	794081	20.90509	-55.69809	43.558	-0.836142
TTA	112767	167626	44736	325129	TAA	75854	101473	148968	326295	39.13986	49.16629	-107.6199	-0.357985
TTC	228489	333895	307656	870040	GAA	328491	167532	382389	878412	-35.90865	66.35582	-21.66033	-0.957647

TTG	194325	409881	130920	735126	CAA	262903	132055	341038	735996	-29.99729	102.5309	-89.04097	-0.118277
TTT	253898	203274	268229	725401	AAA	246485	227564	259556	733605	2.96293	-11.2757	3.286566	-1.124601

Supplementary Information Tables S4. Explanatory notes.

Consensus coding sequence (CCDS) database presents CDS sequences data in fasta format like follows (two sample sequences):

>CCDS1.1|Hs110|chr1
ATGTCCAAGGGGATCCTGCAGGTGCATCCTCCGATCTGCGACTGCCCGGGCTGCCGAATA
>CCDS2.2|Hs110|chr1
ATGGCAGCTGCGGGGAGCCGCAAGAGGCGCCTGGCGGAGCTGACGGTGGACGAGTTCCTA

CCDS annotations on CDS sequences data include strand information like follows (for two sample sequences above):

#chromosome	nc_accession	gene	gene_id	ccds_id	ccds_status	cds_strand	cds_from	cds_to	cds_locations	match_type
1	NC_000001.8	LINC00115	79854	CCDS1.1	Public	-	801942	802433	[801942-802433]	Identical
1	NC_000001.11	SAMD11	148398	CCDS2.2	Public	+	925941	944152	[925941-926012,	Identical

Mono-/di-/tri-nucleotides are counted at all possible positions of CDS, forming a statistics.
Mono-/di-/tri-nucleotides statistics computed for all codons for three codon positions, where possible, separately as well as in total.

For strand-independent case these statistics computed on CDS as they are given in CCDS fasta file, e.g. for two samples above:

ATGTCCAAGGGGATCCTGCAGGTGCATCCTCCGATCTGCGACTGCCCGGGCTGCCGAATA
ATGGCAGCTGCGGGGAGCCGCAAGAGGCGCCTGGCGGAGCTGACGGTGGACGAGTTCCTA

For strand-dependent case these statistics computed on CDS taking into account their strand: CDS on '-' strand reverse complemented
For two samples above sequences input sequencies looks like (first sequence reverse complemented):

TATTCGGCAGCCCGGGCAGTCGCAGATCGGAGGATGCACCTGCAGGATCCCCTTGGACAT
ATGGCAGCTGCGGGGAGCCGCAAGAGGCGCCTGGCGGAGCTGACGGTGGACGAGTTCCTA

For reverse-complemented CDS codon positions keep original semantics, i.e. 1st and 3d codon positions are not interchanged

Mono-/di-/tri-nucleotides statistics collected and compared with reverse complementary counterparts for every codon position and in total.
To compare mono-/di-/tri-nucleotides counts we have used percentage difference formula 200*(A-B)/(A+B).

For two samples above mono- and di-nucleotides statistics with comparison presented below

Mononucleotide CDS counts, strand orientation not taken in account (strand-independent case)

SEQ	P1	P2	P3	TOTAL	RC SEQ	P1	P2	P3	TOTAL	diff P1	diff P2	diff P3	diff
A	10	8	4	22	T	5	12	3	20	66.6667	-40	28.5714	9.5238
C	12	9	13	34	G	13	11	20	44	-8	-20	-42.4242	-25.641
G	13	11	20	44	C	12	9	13	34	8	20	42.4242	25.641
T	5	12	3	20	A	10	8	4	22	-66.6667	40	-28.5714	-9.5238

Mononucleotide Watson (+) strand counts, CDS on Crick (-) strand reverse complemented (strand-dependent case)

SEQ	P1	P2	P3	TOTAL	RC SEQ	P1	P2	P3	TOTAL	diff P1	diff P2	diff P3	diff
A	9	10	4	23	T	6	10	3	19	40	0	28.5714	19.048
C	9	11	13	33	G	16	9	20	45	-56	20	-42.4242	-30.769
G	16	9	20	45	C	9	11	13	33	56	-20	42.4242	30.769
T	6	10	3	19	A	9	10	4	23	-40	0	-28.5714	-19.048

Dinucleotide CDS counts, strand orientation not taken in account (strand-independent case)

SEQ	P1	P2	P3	TOTAL	RC SEQ	P1	P2	P3	TOTAL	diff-P1	diff-P2	diff-P3	diff
AA	2	0	1	3	TT	1	0	0	1	66.6667	#DIV/0!	200	100
AC	1	2	0	3	GT	2	0	2	4	-66.6667	200	-200	-28.571
AG	2	5	1	8	CT	4	2	3	9	-66.6667	85.7143	-100	-11.765
AT	5	1	0	6	AT	5	1	0	6	0	0	#DIV/0!	0
CA	2	1	2	5	TG	3	7	1	11	-40	-150	66.6667	-75
CC	3	1	6	10	GG	3	3	9	15	0	-100	-40	-40
CG	3	5	2	10	CG	3	5	2	10	0	0	0	0
CT	4	2	3	9	AG	2	5	1	8	66.6667	-85.714	100	11.765
GA	4	1	5	10	TC	1	3	2	6	120	-100	85.7143	50
GC	4	7	4	15	GC	4	7	4	15	0	0	0	0
GG	3	3	9	15	CC	3	1	6	10	0	100	40	40
GT	2	0	2	4	AC	1	2	0	3	66.6667	-200	200	28.571
TA	0	2	0	2	TA	0	2	0	2	#DIV/0!	0	#DIV/0!	0
TC	1	3	2	6	GA	4	1	5	10	-120	100	-85.7143	-50
TG	3	7	1	11	CA	2	1	2	5	40	150	-66.6667	75
TT	1	0	0	1	AA	2	0	1	3	-66.6667	#DIV/0!	-200	-100

Dinucleotide Watson (+) strand counts, CDS on Crick (-) strand reverse complemented (strand-dependent case)

SEQ	P1	P2	P3	TOTAL	RC SEQ	P1	P2	P3	TOTAL	diff-P1	diff-P2	diff-P3	diff
AA	1	0	0	1	TT	2	1	0	3	-66.6667	-200	#DIV/0!	-100
AC	2	2	0	4	GT	1	0	2	3	66.6667	200	-200	28.571
AG	5	4	2	11	CT	3	1	2	6	50	120	0	58.824
AT	1	4	1	6	AT	1	4	1	6	0	0	0	0
CA	0	4	4	8	TG	1	6	1	8	-200	-40	120	0
CC	3	2	4	9	GG	4	5	7	16	-28.5714	-85.714	-54.5455	-56
CG	3	4	3	10	CG	3	4	3	10	0	0	0	0
CT	3	1	2	6	AG	5	4	2	11	-50	-120	0	-58.824
GA	5	1	5	11	TC	2	2	1	5	85.7143	-66.667	133.333	75
GC	6	3	6	15	GC	6	3	6	15	0	0	0	0
GG	4	5	7	16	CC	3	2	4	9	28.5714	85.7143	54.5455	56
GT	1	0	2	3	AC	2	2	0	4	-66.6667	-200	200	-28.571
TA	0	1	1	2	TA	0	1	1	2	#DIV/0!	0	0	0
TC	2	2	1	5	GA	5	1	5	11	-85.7143	66.6667	-133.333	-75
TG	1	6	1	8	CA	0	4	4	8	200	40	-120	0
TT	2	1	0	3	AA	1	0	0	1	66.6667	200	#DIV/0!	100
